# The value of “diaphragmatic relaxing incision” for the durability of the crural repair in patients with paraesophageal hernia: a double blind randomized clinical trial

**DOI:** 10.3389/fsurg.2023.1265370

**Published:** 2023-11-10

**Authors:** A. Tsoposidis, A. Thorell, H. Axelsson, M. Reuterwall Hansson, L. Lundell, V. Wallenius, S. Kostic, B. Håkanson

**Affiliations:** ^1^ Department of Surgery, Institute of Clinical Sciences, Sahlgrenska Academy, Sahlgrenska University Hospital, University of Gothenburg, Gothenburg, Sweden; ^2^ Department of Clinical Sciences, Danderyds Hospital, Karolinska Institutet, Stockholm, Sweden; ^3^ Department of Surgery, Ersta Hospital, Stockholm, Sweden; ^4^ Department of Surgery, Institute of Clinical Sciences, Sahlgrenska Academy, Sahlgrenska University Hospital Östra, University of Gothenburg, Gothenburg, Sweden; ^5^ Division of Surgery and Oncology CLINTEC, Karolinska Institutet, Stockholm, Sweden; ^6^ Department of Surgery, Odense University Hospital, Odense, Denmark

**Keywords:** paraesophageal hernia, crural repair, hernia recurrence, diaphragmatic relaxing incision, GERD, obstructive symptoms, quality of life

## Abstract

**Background:**

Surgical repair of paraesophageal hernias (PEHs) is burdened with high recurrence rates, and hitherto various techniques explored to enforce the traditional crural repair have not been successful. The hiatal reconstruction in PEH is exposed to significant tension, which may be minimized by adding a diaphragmatic relaxing incision to enhance the durability of the crural repair.

**Patients and methods:**

All individuals undergoing elective laparoscopic repair of a large PEH, irrespective of age, were considered eligible. PEHs were classified into types II–IV. The preoperative work-up program included multidetector computed tomography and symptom assessment questionnaires, which will be repeated during the postoperative follow-up. Patients were randomly divided into a control group with crural repair alone and an intervention group with the addition of a left-sided diaphragmatic relaxing incision at the edge of the upper pole of the spleen. The diaphragmatic defect was then covered by a synthetic mesh.

**Results:**

The primary endpoint of this trial was the rate of anatomical PEH recurrence at 1 year. Secondary endpoints included symptomatic gastroesophageal reflux disease, dysphagia, odynophagia, gas bloat, regurgitation, chest pain, abdominal pain, nausea, vomiting, postprandial pain, cardiovascular and pulmonary symptoms, and patient satisfaction in the immediate postoperative course (3 months) and at 1 year. Postoperative complications, morbidity, and disease burden were recorded for each patient. This was a double-blind study, meaning that the operation report was filed in a locked archive to keep the patient, staff, and clinical assessors blinded to the study group allocation. Blinding must not be broken during the follow-up unless required by any emergencies in the clinical management of the patient. Likewise, the patients must not be informed about the details of the operation.

**Trial Registration:**

ClinicalTrials.gov, identification number NCT04179578.

## Introduction

Paraesophageal hernias (PEHs) are rare, accounting for less than 5% of all hiatus hernias (HHs) (1), and occur most commonly in older people. A PEH is characterized by a herniation of the gastric fundus and sometimes the entire stomach (with or without additional viscera) through a widened diaphragmatic hiatus. The indications for surgical repair are controversial but must balance the patient's fitness for surgery and respective symptom burden (2–6). Surgical repair traditionally includes dissection of the hernia sac from the mediastinum, reduction of herniated intra-abdominal organs, posterior repair of the crura (with or without mesh), and a fundoplication to control reflux.

Although good clinical outcomes have been reported with direct suturing of the hiatus, clinical and/or radiological recurrences have been described in up to 60% of patients (7–12). The use of mesh to reinforce the crura has been suggested to reduce the recurrence of PEH based on the same principles that have been successfully used in groin hernia repair. However, although the use of mesh in laparoscopic surgery of hiatus hernia has increased, the indications for mesh reinforcement can be seriously questioned. The primary concern regarding the use of mesh is the long-term risk of mesh erosion into the esophagus and other adjacent vital structures. To avoid or reduce this risk, the synthetic mesh has been abandoned in favor of biological meshes (6, 13–18). However, results from more recent reports suggest that the long-term durability of mesh (irrespective of type) and suture cruroplasty did not differ. Indeed, the same high recurrence rates have been reported during the first 3–5 years postoperatively in patients with smaller hiatus hernias, including the traditional sliding hernias (type I HH) (6, 19–24). Accordingly, novel approaches must be explored to enable more durable reconstructions in HH repair.

One possible explanation behind the high recurrence rates can be found in the three-dimensional structures of the hiatus, which are in constant motion. In addition, the counteracting pressures prevailing in the abdominal and chest cavities create a situation where the experiences from other types of hernia repair do not apply (10, 24–26). However, a complementary explanation is that during laparoscopic repair there may be an underappreciation of the tension applied to the repair. This tension comes from two major directions: axial tension related to esophageal shortening and lateral tension related to the widely separated crura that must be reapproximated as part of the repair. The pathogenetic relevance of this lateral tension can conceptually be addressed by adding diaphragmatic relaxing incisions to enhance the durability of the crural repair (27, 28). Until now, this concept has not been evaluated in the randomized clinical trial setting.

## Objectives

In this trial, we compared the rates of anatomical PEH recurrence at 1 year in patients allocated to diaphragmatic relaxing incisions to those with a crural repair alone.

Secondary objectives included rates of symptomatic gastroesophageal reflux disease (GERD), dysphagia, odynophagia, gas bloat, regurgitation, chest pain, abdominal pain, nausea, vomiting, postprandial pain, cardiovascular and pulmonary symptoms, and patient satisfaction in the immediate postoperative course (3 months) and at 1 year. The Clavien–Dindo classification and the comprehensive complication index were also used to measure postoperative complications, morbidity, and disease burden for each patient (29, 30).

## Patients and methods

The trial occurred at two high-volume centers, one in Stockholm and one in Göteborg, Sweden. All operations were performed by or directly supervised by one experienced upper gastrointestinal surgeon, and all procedures were completed within a university teaching hospital setting. All individuals undergoing elective laparoscopic repair of a PEH, irrespective of age, were considered eligible. PEHs were classified into type II (pure paraesophageal), type III (combined sliding and paraesophageal), and type IV (combined with other hernia sac contents than the stomach). Patients who have undergone previous surgery involving the stomach or the esophagogastric junction or who require any additional procedure affecting the hiatal hernia (HH) repair were excluded.

Pre- and postoperative investigations included esophagogastroduodenoscopy, multidetector computed tomography (MDCT), and symptom assessment questionnaires. Esophageal high-resolution manometry and pH monitoring were used selectively in patients with significant reflux symptoms but most often omitted in patients in whom the indication for surgery is mechanical symptoms emanating from the hernia.

## Trial group allocation

Patients were asked to sign an informed consent before surgery and were randomly divided into one of the two groups in a 1:1 ratio during surgery, after the induction of the anesthesia:

Group 1 (control group) patients had a crural repair using sutures alone (control) + a total fundoplication.

**Figure 1:**
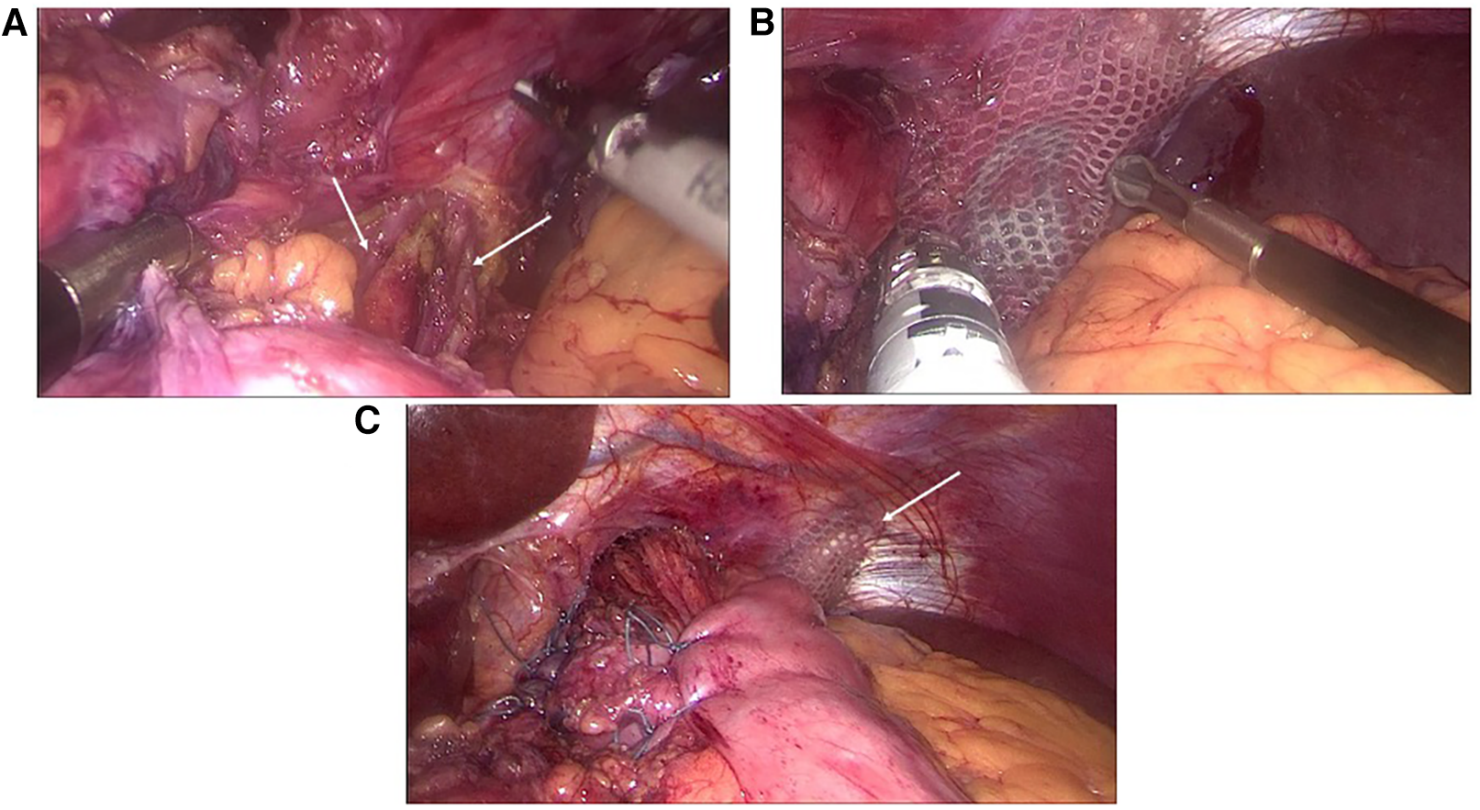
Intraoperative images demonstrating (**A**) the left-sided lateral diaphragmatic incision (also marked by the arrows) originating from the costal area close to the pole of the spleen, (**B**) the application of the mesh to cover the diaphragmatic defect, and (**C**) an overview of the end result including the total fundoplication.

Group 2 (intervention group) was the same as group 1 but before adapting the crura, a left-sided 5–6-cm-long diaphragmatic relaxing incision was added at the edge of the upper pole of the spleen (Figure 1).

## Randomization and blinding

Randomization was performed during the operation by opening a sealed envelope. The envelopes were prepared before the start of the trial and shuffled independently by research nurses. More envelopes were prepared than needed to ensure that the operating surgeon cannot anticipate the randomization. A computer-generated randomization list in blocks of eight was used. The subsequent operation report, with information on the specific type of repair performed, was not included in the digital patient chart. Instead, a hard copy was created and kept in a sealed envelope, which is filed in a locked archive to keep the patient, staff, and clinical assessors blinded to the study group allocation. Blinding must not be broken during the follow-up unless required by any emergencies in the clinical management of the patient. Likewise the patients will not be informed about the details of the operation. The same is true for the clinical follow-up investigators.

## Operating technique

Before commencing the trial, surgical techniques were standardized and harmonized across the two sites after a consensus meeting between the participating surgeons and an exchange of videos of the standard operating techniques.

The initial steps entailed full dissection and resection of the hiatus hernia sac from the mediastinum, and complete reduction of the sac's contents into the abdomen. An esophageal lengthening procedure was not added. Five trocars were utilized. Ultracision (Ethicon Endo-Surgery) was used for dissection. After completing the hernia sac resection, the anatomical preparation of both crural muscles followed. Mobilization of the mediastinal esophagus was completed to allow at least 3 cm of the lower esophagus to rest without tension below the hiatus. The posterior and anterior branches of the vagal nerve were identified and kept within the wrap. The gastric fundus was mobilized as judged necessary to complete a floppy full wrap, with a wrap length of 2 cm by three interrupted non-absorbable sutures (GORE-TEX or Ethibond), all anchored to the esophageal muscular wall. No formal bougie was used in the esophagus during the procedure. In the suture-alone group, the right and left crura were approximated posteriorly to the esophagus using at least three continuous stitches with non-absorbable sutures (GORE-TEX or Ethibond) and a suture-to-suture distance of 5–8 mm, leaving an appropriate remaining opening for the esophagus.

In the intervention group, a left-sided diaphragmatic relaxing incision was added at the edge of the upper pool of the spleen before adapting the crura (Figure 1). The length of this incision was 5–6 cm. After the completion of the crural sutures, the gap in the diaphragm was covered by a synthetic mesh, which was anchored to the remaining diaphragm by staples.

During the procedure, the esophageal length was measured endoscopically before hernia sac reduction and esophageal mobilization and at the time point after completion of the hiatal reconstruction. The length from the incisors to the GEJ was recorded in centimeters. The hiatal area was calculated before reconstruction by measuring (a) the length of the right crus and (b) the distance between the base of the left crus and the base of the right crus. The following formula was used for the calculation of the hiatal area (*A*, in cm^2^):

$$\mathrm{Hiatal}\;\mathrm{area}\;A:\;A=\frac{a\sqrt{4b^{2}-2a^{2}}}{4}$$

## Statistics and sample size

Based on the assumption that the 12-month CT detected recurrence rate is at least 30%, it can be estimated that, by the effect of the intervention, these figures were reduced by 50%–15%. Accordingly, by enrolling 35 patients into each group, such a difference can be detected at a probability of 5% and a power of 80%. To compensate for the loss of follow-up and early exit of patients, 80 patients were enrolled.

All data were entered into a computerized database. Analyses were performed on an intention-to-treat basis with patients analyzed according to randomization. The two groups were compared separately. The *χ*^2^ test was used to evaluate 3 × 2 contingency tables. Comparison of continuous data sets was completed using one-way analysis of variance. A *p*-value of <0.05 was considered statistically significant. The statistical analyses were performed using the statistical software package SPSS, version 26.0 (SPSS, Inc., Chicago, IL, USA).

## Follow-up assessment

After discharge, the patients were followed up at 3 and 12 months. The Swedish version of the validated Short Form-36 (SF-36) questionnaire was used for global QoL assessment, and data were then presented as physical and mental component scores (PCS and MCS). For each subscale score (0–100), higher values reflect improved health status. The gastrointestinal symptom rating scale (GSRS) is a validated questionnaire containing five dimensions of abdominal symptoms (gastroesophageal reflux, abdominal pain, indigestion, obstipation, and diarrhea). The subscales were presented according to a seven-point Likert scale, and the mean item scores of the respective domain were used throughout the study. Higher values represent more severe symptoms. For these two questionnaires, comparative values for the adult normal population were available. In addition, a standardized specific questionnaire was used, which includes a four-graded scale to describe any dysphagia for solid and liquid food components (31). The MDCT was done only at 12 months postoperatively.

## Discussion

The number of PEH repairs has increased significantly over the last three decades, but the optimal technique for hiatal closure, either sutured or mesh-augmented (absorbable or non-absorbable), remains controversial. In the 1990s, the standard laparoscopic approach was developed to include PEH repair: during these operations, the need to dissect the entire hernia sac from the mediastinum and restore the anatomy in the GEJ was emphasized. Despite these many precautions, at least a third of these patients develop a radiological hernia recurrence by 5 years postsurgery, albeit most patients probably remain asymptomatic (8, 24). To address the problem of high recurrence rates, tension-free mesh-augmented hernioplasty, analogous to that used to repair groin and abdominal wall hernias, has been proposed. Synthetic absorbable and non-absorbable meshes for hiatal closure have now been evaluated in randomized controlled trials. Data from recent meta-analyses concluded that using mesh in HH repair does not offer any advantage over sutured hiatal closure alone (23). As both techniques deliver good and comparable clinical outcomes, a suture-only technique remains an appropriate standard of care surgical approach.

Nevertheless, hernia recurrence remains the Achilles’ heel of PEH repair. Recurrence after laparoscopic repair seems to be higher than in previous reports on open repairs (1, 10, 24). The explanation for the higher recurrence rate with laparoscopic repair remains unclear, but explanatory factors may include the lack of deep bites during crural closure, with the use of laparoscopic suturing devices, and fewer adhesions associated with laparoscopy compared with an open procedure. However, an alternative explanation may be that during laparoscopic repair, the applied tension may be underappreciated during the repair. The consequences of tension on hernia recurrence are well documented in other settings, including inguinal and ventral hernia repair (32). To reduce tension and improve outcomes with laparoscopic HH repair, adjunct techniques have been adopted. These include a diaphragm relaxing incision, esophageal lengthening wedge-fundectomy, or traditional Collis gastroplasty (1, 4, 27, 28). A diaphragmatic relaxing incision has rarely been tested and, when so, most commonly executed on the right side. This is the easiest approach for the diaphragmatic relaxing incision. However, the right-sided relaxing incision may often be inadequate since the right crus might be frail and phylogenetically only a part of the left. Therefore, a left-sided diaphragmatic relaxing incision carries potential advantages and can almost always be employed. A phrenic nerve injury must be avoided when carrying out a left-sided incision. Large openings (5–6 cm) between the abdomen and thorax are well tolerated during laparoscopic surgery, and in the absence of an injury to lung parenchyma, no chest tube or pleural drainage catheter is routinely placed at the end of the procedure. The follow-up MDCT investigation at 12 months was conducted to determine whether any local complications can be detected at the incision site covered by a synthetic mesh placed at a safe distance from the hiatal region.

The results from the very few retrospective studies that have explored the options offered by these approaches (i.e., either esophageal lengthening or diaphragm relaxing incision) addressing the two major tension forces operating on the crural repair do not offer much guidance in the clinical decision-making process to minimize the risk for recurrent HH. Well-designed, separate RCTs with simple crural closure as reference/control must be completed before alternative recommendations can be made in the surgical management of PEH.

## Ethics statement

The studies involving humans were approved by the Human Research Ethics Committee and the Clinical Research Ethics Committees (reference number 2019-02801). The studies were conducted in accordance with the local legislation and institutional requirements. The participants provided their written informed consent to participate in this study.
